# Second Line Treatment Decision as Per Standard of Care or Foundation Medicine in Patients With Locally Advanced or Metastatic Non‐Small Cell Lung Cancer

**DOI:** 10.1002/cam4.71790

**Published:** 2026-04-12

**Authors:** Federico Rojo, Enriqueta Felip, Oscar Juan Vidal, Rosario García Campelo, David Aguiar Bujanda, Josefa Terrasa Pons, Rafael López‐Castro, Antonio Calles, Alfredo Paredes Lario, Reyes Bernabé Caro, Isidoro Barneto Aranda, Luis García‐Palacios, Javier De Castro Carpeño

**Affiliations:** ^1^ IIS‐Hospital Universitario Fundación Jiménez Díaz‐CIBERONC Madrid Spain; ^2^ Vall D'hebron University Hospital Barcelona Spain; ^3^ Hospital Universitari i Politécnic La Fe Valencia Spain; ^4^ University Hospital A Coruña Coruña Spain; ^5^ Hospital Universitario de Gran Canaria Doctor Negrín Las Palmas De Gran Canaria Spain; ^6^ Hospital Son Espases Mallorca Spain; ^7^ Hospital Clínico Universitario de Valladolid Valladolid Spain; ^8^ Hospital General Universitario Gregorio Marañón Madrid Spain; ^9^ Hospital Universitario Donostia San Sebastián Spain; ^10^ Hospital Universitario Virgen del Rocío Sevilla Spain; ^11^ Hospital Universitario Reina Sofía Córdoba Spain; ^12^ Medical Department Roche Farma Madrid Spain; ^13^ Hospital Universitario La Paz, IdiPAZ Madrid Spain

## Abstract

**Background:**

Molecular genetic profiling is crucial for treatment choice in patients with advanced non‐small cell lung cancer (NSCLC). The Lung‐ONE study aimed to determine the clinical utility of comprehensive genomic profile (CGP) tests, such as FoundationOne CDx or FoundationOne Liquid, in guiding second line therapy decisions for advanced NSCLC patients in Spain. Additionally, the study sought to describe the genomic alterations found in these patients.

**Methods:**

This national, multicenter, prospective study included adult patients diagnosed with advanced/metastatic NSCLC undergoing first line treatment with molecular diagnostic wild‐type (or unknown) for at least ALK, EGFR, and ROS‐1 genes under clinical practice conditions. Physicians planned their therapeutic decision for second line treatment according to the standard of care (SOC) and subsequently re‐evaluated this initial decision after a Foundation One or Foundation ACT test (FMI) report was provided. Patients continue to be followed up for up to 2 years.

**Results:**

The study analyzed 151 advanced NSCLC patients. The FMI report identified 542 driver mutations in actionable genes for 132 patients, including mutations in EGFR, ALK, and BRAF that were previously missed by single‐gene testing as per SOC. Additional driver alterations were also detected in ERBB2/HER2, MET, and RET genes. FMI identified mutations with designated therapies in 116 patients. Consequently, clinicians identified 44 (29.1%) patients with gene alterations associated with off‐label drugs, and 13 (8.6%) patients were directed toward clinical trials.

**Conclusions:**

Using FMI data, clinicians were able to direct patients toward clinical trials and to modify SOC clinical management for several NSCLC patients. These results demonstrate the benefit of FMI genomic profiling in identifying actionable driver mutations that would otherwise be missed by SOC methodology. The findings suggest that CGP is a promising and robust tool for improving personalized medicine in NSCLC treatment in Spain.

## 1 Introduction

In 2022, lung cancer emerged as the leading cause of cancer‐related morbidity and mortality globally, with nearly 2.5 million new cases and over 1.8 million deaths. Although incidence declined in the US and EU due to reducing smoking prevalence [1], it remained the top cancer in men, and second in women [2]. About 85% of lung cancer cases are non‐small cell lung cancer (NSCLC) [3].

NSCLC treatment depends on histological classification, stage, and genetic profiling, including surgery, radiotherapy, chemotherapy, immunotherapy or targeted therapies [4, 5]. Prevailing clinical practice for patients with locally advanced (stage III) NSCLC is to check its suitability for resection surgery. Unresectable stage III NSCLC cases receive concurrent or sequential chemoradiotherapy [4, 5]. For unresectable stage III NSCLC patients that are not candidates for radiotherapy as first line treatment and for metastatic (stage IV) NSCLC patients, genetic profiling will determine the choice of treatment [4].

Molecular testing and personalized oncology aim to match patients precisely to treatments with the highest potential of success. Specific gene alterations or mutations can be targeted for treatment, may predict better or worse survival outcomes, and can provide valuable information for alternative treatment options [6]. The current Clinical Practice Guidelines of the European Society for Medical Oncology (ESMO) guidelines are in line with the National Comprehensive Cancer Network (NCCN) Clinical Practice Guidelines in Oncology (2022) and the American Society of Clinical Oncology (ASCO) guidelines (2022 and last update in 2024 for stage IV NSCLC) for the recommended biomarkers to test in advanced NSCLC to guide clinical decisions [7, 8, 9, 10, 11]. Patients with genomic alterations listed as level I according to ESMO Scale for Clinical Actionability of molecular Targets (ESCAT) [12, 13, 14] must have access to treatment as per standard of care (SOC). The main alterations to highlight include EGFR (exon 20 insertions and mutations), KRAS (G12C), BRAF (V600E), MET (exon 14 skipping), HER2 (exon 20 mutations), fusions for ALK, RET, NRTK and ROS1, and the detection of PDL‐1 expression [13, 14, 15].

In Spain, biomarker testing is conducted according to guidelines set by the Spanish Society of Medical Oncology (SEOM) and the Spanish Society of Pathology (SEAP) [5, 16, 17], which primarily focus on identifying alterations in a limited number of molecular markers. The accessibility to the corresponding genetic tests depends on several factors, from quality and grade of approval of the methodology, to the region where the center is located. This scenario translates into an enormous heterogeneity regarding both the genetic information available for the physicians and their decision for a first‐ or second‐line therapy according to this information. The scarcity of tumor tissue to carry out all required tests and sequential molecular testing, often employed in routine clinical practice, is associated with higher costs and longer turnaround time [18]. Next‐generation sequencing (NGS) allows simultaneous testing of multiple somatic genetic alterations in a short time frame, saving tissue samples and time, while being more affordable. Indeed, NGS remains the recommendation in daily practice when assessing tumor somatic genetic alterations for advanced NSCLC [13].

Unlike most in‐house genomic NGS panels used within the SOC in oncology, even those using NGS, Foundation Medicine (FMI) is a “Comprehensive Genomic Profiling” (CGP) test, able to detect cancer‐relevant genes with all classes of alterations across the entire coding region, with validated performance [19]. Moreover, FMI is a hybridization capture‐based NGS method which does not miss any relevant gene or genetic alteration [20, 21]. As for relevant NSCLC‐related somatic genomic alterations, this study focuses on driver alterations, which contribute causally to tumor initiation, progression, or maintenance, and on actionable alterations, defined as those with current or emerging therapeutic implications, including approved drugs, guideline‐supported off‐label uses, or clinical trials specifically targeting the alteration [12, 22]. FMI technology can identify molecular alterations otherwise missed by SOC molecular panels. Considering the limited patient genetic information available for the clinicians within standard clinical practice, a CGP provided by FMI might help physicians to calibrate their decisions on second line treatments for advanced NSCLC adenocarcinoma in Spain.

The LungONE study had as primary objective to describe the clinical management of second line SOC treatment in patients with locally advanced/metastatic NSCLC with adenocarcinoma histology, when comprehensive genomic profile information, based on a Foundation One or Foundation ACT test, was provided. The secondary objectives for this study were to describe the number and type of actionable molecular aberrations and evaluate treatment decisions according to FMI report and SOC panels.

## 2 Methods

### 2.1 Study Design and Patients

This was a national, multicenter, prospective study conducted in patients diagnosed with NSCLC with adenocarcinoma histology who were receiving or had just received their first line treatment under clinical practice conditions.

The study was conducted at the medical oncology departments of 12 hospitals in Spain. The participating investigators enrolled consecutive patients aged ≥ 18 years, diagnosed with locally advanced or metastatic NSCLC with adenocarcinoma histology (i.e., stage IIIB not eligible for definitive chemoradiation therapy, stage IV, or recurrent as it is classified by the American Joint Committee on Cancer [AJCC], 8th edition), who completed the diagnostic molecular panel (biomarker/gene) as per SOC at the site including, at least, the ALK, EGFR and ROS‐1 markers, who were receiving or had just received first line treatment, had measurable disease, ECOG performance status (PS) of 0, 1, or 2 and life expectancy ≥ 12 weeks. Patients who were receiving or previously had received any other treatment with targeted therapy including immunotherapy and with malignancies other than NSCLC were to be excluded. The study inclusion period was 9 months (October 2018 and July 2019). Patients were followed up for a maximum of 24 months since the last patient was included in the study.

### 2.2 Compliance With Ethical Guidelines

The study is in accordance with the Declaration of Helsinki and Good Clinical Practice Guidelines and applicable regulatory requirements. The study protocol was approved by the Independent Ethics Committee (IEC) of Hospital Universitario de Gran Canaria Dr. Negrín (CEIm code 2018‐185‐1). Written informed consent was obtained from all patients to participate in the study before their enrolment.

### 2.3 Samples

The same diagnostic sample was used for both SOC and FMI analyses, primarily obtained through surgical resection or fine needle aspiration. Genetic profiling via CGP was planned during first‐line treatment. For patients lacking these tissue samples or when certain markers could not be characterized due to tissue depletion, liquid biopsies were collected.

In terms of quality control, SOC testing varied by site: EGFR was analyzed via Sanger sequencing, pyrosequencing, NGS, or qPCR with validated commercial kits; ALK and ROS1 were detected using PCR, NGS, NanoString, IHC, or FISH; PD‐L1 assessment relied solely on IHC with specified antibodies; and BRAF was tested by pyrosequencing or qPCR.

Quality control for FMI genetic profiling samples involves pathology review of all specimens to confirm they meet strict analysis criteria, such as sufficient tissue volume and appropriate preservation. Additional controls evaluate DNA quantity and quality extracted from FFPE samples, alongside metrics for library preparation and sequencing performance, ensuring only samples that meet predefined standards enter the customized analysis pipeline for reliable genomic profiling. The percentage of tumor cells, calculated as tumor nuclei divided by total nucleated cells, should optimally be at least 30% for solid tumors, with a minimum threshold of 20% for acceptance; liver specimens require even higher percentages due to their polyploid nuclei. Sample quality is evaluated based on tissue preservation, nucleated cell density, absence of excessive necrosis or fibrosis, and appropriate fixation; suboptimal or poorly enriched samples may yield only conditional results or require additional tissue submission. Quality assurance also included external proficiency schemes (SEAP, UKNEQAS, NordiQ, EQA, MNQ), analytical validation of key biomarkers, CE‐mark verification of reagents, standardized test protocolization, and use of internal positive/negative controls. For FMI testing, strict sample input criteria ensured validated FDA‐approved performance [23].

### 2.4 Foundation Medicine

Foundation Medicine (FMI) is an FDA‐approved NGS‐based in vitro diagnostic device with several formats validated in top tier peer‐reviewed journals, including “FoundationOne CDx” for solid tumors (F1CDx) and “FoundationOne Liquid CDx” (F1LCDx), both used in the samples from the present study [20, 24]. It is performed exclusively by Foundation Medicine as a central laboratory, using DNA from cancer patients either extracted from formalin‐fixed paraffin‐embedded tumor samples or circulating cell‐free DNA isolated from plasma.

As previously described [25, 26], all coding exons of 309 genes are targeted; select intronic or non‐coding regions are targeted in 21 of these genes. Additionally, select intronic or non‐coding regions are targeted in 15 genes, resulting in 324 total targeted genes. Sequence data are processed using a custom analysis pipeline that filters sequencing artifacts and variants known to be benign. Known and likely pathogenic variants implicated in cancer are reported, which may be somatic and/or germline variants. The assay detects substitutions, indels, genomic rearrangements, copy number alterations (CNAs; amplifications and losses), and tumor fraction (TF) [21, 27, 28, 29]. Moreover, these tests also determine the genetic signature of the tumor, by providing microsatellite status (MSI), and tumor mutational burden (TMB), validated with promising results as a biomarker for response to immunotherapy [30, 31]. MSI status is determined by genome wide analysis of 95 microsatellite loci.

### 2.5 Study Outcomes

The primary objective for this study was to describe the clinical management of second line SOC treatment in patients with locally advanced/metastatic NSCLC with adenocarcinoma histology, when comprehensive genomic profile information, based on a Foundation One or Foundation ACT test (FMI), was provided.

The present article shows results for the following secondary objectives: (i) to evaluate if there was any change in previously planned second line treatment decisions after receiving the FMI report in patients with locally advanced or metastatic NSCLC, (ii) to describe the patient status 2 years after the last patient has been included in the study, (iii) to describe the number and type of actionable molecular aberrations (i.e., biomarker/gene) detected.

### 2.6 Statistical Analysis

The sample size was determined to achieve the primary objective, ensuring that secondary objectives are also met. To describe clinical management and to detect treatments used in at least 20% of patients, considering four main second‐line therapies (chemotherapy, radiotherapy, chemoradiation therapy, targeted therapy, inclusion in clinical trials, and others), the study required 171 patients. With a 95% confidence interval and 6% precision, and accounting for a 5% loss rate, the total sample size was rounded to 180 patients.

Quantitative variables were described using measures of central tendency and dispersion (mean, standard deviation, median, minimum, maximum, first quartile [Q1] and third quartile [Q3]) and the results were expressed as mean ± standard deviation (SD) or median (range Q1–Q3). The distribution of absolute and relative frequencies was described for the qualitative variables. The 95% confidence intervals (95% CI) were shown for the main outcome variables. No imputations were conducted about absent data, which were left as missing values.

## 3 Results

### 3.1 Demographic, Clinical and NSCLC Characteristics of Patients

**FIGURE 1:**
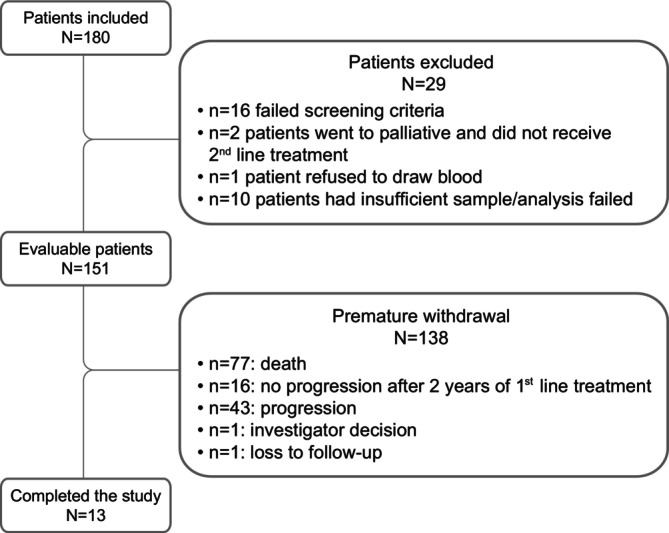
Patient flowchart. 1st = first; 2nd = second lines of treatment.

A total of 180 patients were enrolled in the study between October 2018 and July 2019. Of these, 29 patients were excluded for not fulfilling the screening criteria, leaving 151 evaluable patients (Figure 1). At study completion, 138 (91.4%) patients had withdrawn due to: death (*n* = 77, 55.8%), progression to next line therapy (*n* = 43, 31.2%), no progression after 2 year of first line treatment (*n* = 16, 11.6%), lost to follow‐up (*n* = 1, 0.7%), or following investigator's decision (*n* = 1, 0.7%).

The mean (SD) age of patients was 62.4 (9.4) years, with 62.3% males (Table 1). Regarding patient smoking habits, 13.9% were non‐smokers, 53% ex‐smokers, 21.9% smokers, and 11.3% active smokers. Most patients (90.7%) had ECOG PS of 0 or 1. Overall, 80.1% of patients had at least one comorbid condition (Table 1), with cardiovascular system (38.4% of patients) and endocrine/metabolic system (29.8% of patients) being the most frequent at study start.

**TABLE 1:** Baseline sociodemographic and clinical characteristics.

Characteristic	*N* = 151
*Sociodemographic characteristics*	
Age (years), mean ± S.D.	62.4 ± 9.4
Gender, male, *n* (%)	94 (62.3)
Body surface (m^2^), mean ± S.D.	1.8 ± 0.2
*Clinical characteristics*	
Smoking habit, *n* (%)	
No smoker	21 (13.9)
Ex‐smoker	80 (53.0)
Smoker	33 (21.9)
Active smoker	17 (11.3)
Relevant comorbidities	
At least one comorbidity, *n* (%)	121 (80.1)
Organ system most frequently affected, *n* (%)	
Cardiovascular system	58 (38.4)
Endocrine/metabolic system	45 (29.8)
Respiratory system	37 (24.5)
ECOG performance status, *n* (%)	
ECOG 0	53 (35.1)
ECOG 1	84 (55.6)
ECOG 2	14 (9.3)

*Note:* Smoking habits are classified as follows: “No smoker” if patient had smoked < 100 cigarettes lifelong; “Ex‐smoker” if patient has left smoking for more than 1 year; “Smoker” if patient does not smoke but has left smoking for less than 1 year; “Active smoker” if patient is still smoking.

The median time since NSCLC diagnosis to study inclusion was 7.2 (range 0.5–140.6) months and to start first line treatment was 1.4 (range 0.1–121.2) months (Table 2). Tumors with reported location were in the right side in 101 (67.3%) and in the left side of the lung in 49 (32.7%) patients. At study entry, 69.6% patients had primary tumor with a clinical classification equal to or higher than T2, 65.6% had regional lymph node metastases (N1–N3), and 85.5% had distant metastasis (M1–M1c), with a consequent stage equal to or higher than IIIB disease. Metastatic lesions at the start of the study were mainly located in the lung or pleura (40.4%), bone (19.9%), and lymph nodes (11.9%).

**TABLE 2:** NSCLC characteristics.

Data related to NSCLC cancer	
Time since diagnosis to study inclusion (months), median (range)^a^	7.2 (0.5–140.6)
Time since diagnosis to 1st line treatment (months), median (range)^b^	1.4 (0.1–121.2)
Tumor lung site	
Right side, *n* (%)^c^	101 (67.3)
Upper, *n* (%)^e^	61 (60.4)
Middle, *n* (%)^e^	13 (12.9)
Lower, *n* (%)^e^	27 (26.7)
Left side, *n* (%)^c^	49 (32.7)
Upper, *n* (%)^d^	30 (61.2)
Lower, *n* (%)^d^	19 (38.8)
Cancer TNM staging	
Primary tumor, *n* (%)^f^	
Tx	27 (17.9)
T1	3 (2.0)
T1a	2 (1.3)
T1b	6 (4.0)
T1c	8 (5.3)
T2	11 (7.3)
T2a	16 (10.6)
T2b	14 (9.3)
T3	22 (14.6)
T4	42 (27.8)
Local lymph nodes, *n* (%)^f^	
Nx	27 (17.9)
N0	25 (16.6)
N1	20 (13.2)
N2	46 (30.5)
N3	33 (21.9)
Distant metastasis, *n* (%)^f^	
M0	22 (14.6)
M1	27 (17.9)
M1a	37 (24.5)
M1b	19 (12.6)
M1c	46 (30.5)
Stage, *n* (%)^g^	
IA1	1 (0.7)
IIA	2 (1,3)
IIIA	3 (2)
IB	3 (2)
IIB	3 (2)
IIIB	7 (4.7)
IIIC	1 (0.7)
IV	27 (18.1)
IVA	56 (37.6)
IVB	46 (30.9)
Location of metastasis, *n* (%)^f^	
Lung/pleura	61 (40.4)
Bone	30 (19.9)
Lymph node	18 (11.9)
Brain	16 (10.6)
Liver	6 (4.0)
Other^h^	20 (13.2)

^a^ Excluded data: *n* = 1.

^b^ Excluded data: *n* = 4.

^c^ Missing data: *n* = 1, percentages calculated over a population *n* = 150.

^d^ Percentages calculated over the total number of patients who had metastasis in the left lobe (*n* = 49).

^e^ Percentages calculated over the total number of patients who had metastasis in the right lobe (*n* = 101).

^f^ Percentages calculated over the total population (*n* = 151).

^g^ Excluded data: *n* = 2, percentages calculated over a population *n* = 149.

^h^ Other (including adrenal gland, suprarenal, pericardium, stomach, mediastinum, and subcutaneous).

### 3.2 NSCLC First Line Treatments

First‐line NSCLC therapies comprised 384 treatments, where 279 (72.7%) were for induction and 105 (27.3%) for maintenance. A higher percentage of patients received folic acid antagonists (*n* = 172; 44.8%) followed by platinums (*n* = 138; 35.9%; Table S1 in the electronic Supporting Information). A total of 40 (26.5%) patients underwent surgery and 35 (23.2%) patients received a mean of 9.1 cycles of radiotherapy.

Complete response was achieved in 2 (1.3%) patients, 62 (41.6%) patients had partial response, and in 55 (36.9%) patients the disease was stable during the first line treatment (Figure S1 in the electronic Supporting Information).

### 3.3 Genetic Profiling as Per SOC

**FIGURE 2:**
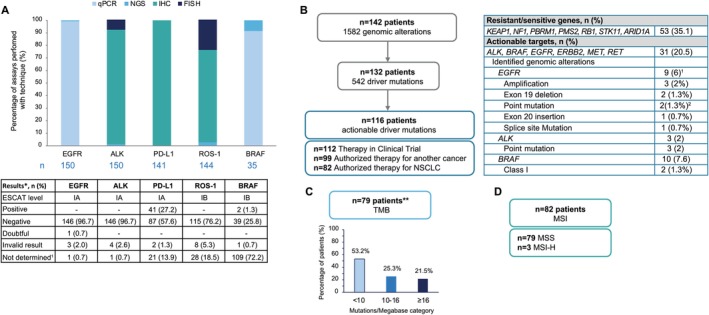
Genetic profiling. (A) Genetic profiling performed as per SOC. (B) Genetic profiling performed by FMI comprehensive genomic profiling. (C) TMB analyzed by FMI. (D) MSI analyzed by FMI. *EGFR‐activating mutations, ALK rearrangements, ROS‐1 fusion, BRAF V600E mutation, PD‐L1 detection. **Missing data *n* = 72. ^1^8 patients have registered one mutation in EGFR and 1 patient has registered 2 mutations in EGFR. ^2^1 patient has registered 1 point mutation, and 1 patient has registered 2 point mutations in EGFR. CGP, comprehensive genomic profiling; IHC, immunohistochemistry; FISH, fluorescence in situ hybridization; FMI, foundation medicine information genetic CGP; MSI, microsatellite instability; MSI‐H, MSI high; MSS, microsatellite stable; NSCLC, non‐small cell lung cancer; NGS, next generation sequencing; SOC, standard of care; qPCR, quantitative polymerase chain reaction; TMB, tumor burden.

Overall, tissue biopsies were collected from 150 (99.3%) patients and liquid biopsies from 3 (2%) patients. More than 90% of patients were tested for EGFR, ALK, ROS‐1, and PD‐L1 as per standard of care, and 35 patients (23.2%) were tested for BRAF alterations (Figure 2). EGFR and BRAF were primarily analyzed using qPCR (99.3% and 91.4%, respectively), while ALK, ROS‐1, and PD‐L1 were predominantly evaluated using IHC (91.3%, 73.6%, and 93.4%, respectively).

The SOC genetic tests did not detect EGFR‐activating alterations, ALK rearrangements, and ROS‐1 fusion (Figure 2). Two (1.3%) patients had BRAF V600E mutations. Samples from 41 (27.2%) patients scored positive for PD‐L1, 87 (57.6%) were negative, and samples from 2 (1.3%) patients were invalid.

Additional mutations were found in 8 patients, including mutations in BRAF, KRAS, STK11, and TP53 (Table S2 in the electronic Supporting Information).

A comparison between FMI testing through liquid and tissue biopsy has been performed (Table S3). Therapeutical orientations were significantly more common in cases assessed by tissue analysis (88.2%) than by blood analysis (53.1%, *p* = 0.001). Most patients in both groups were included in clinical trials (97.8% tissue, 92.3% blood). Authorization of therapies specifically for NSCLC was also more frequent in the tissue group (76.7%) than in the blood group (50.0%).

### 3.4 Genetic Profiling by Foundation Medicine

A total of 102 (67.5%) tissue samples for Foundation One analysis and 49 (32.5%) blood samples for Foundation One Liquid were collected. The FMI report identified a total of 1582 genomic alterations in 142 patients. From these alterations, 542 (34.3%) were in actionable genes and were found in 132 patients (Figure 2). Actionable driver mutations with therapeutic orientations were confirmed for 116 patients; 112 therapies were in clinical trials, and 82 were authorized therapies for NSCLC.

Alterations in genes associated with resistance or sensitivity, including KEAP1, NF1, PBRM1, PMS2, RB1, STK11, and ARID1A, were identified in 53 patients (35.1%) (Figure 2). For actionable genes, alterations were found for ESCAT level IA (ALK and EGFR), IB (BRAF and MET), IC (RET), and IIB (ERBB2/HER2) in 31 patients (20.5%). Among the 9 patients (6%) showing genomic alterations in the EGFR gene, 3 exhibited gene amplifications, 2 had exon 19 deletions, and 1 patient registered an exon 20 insertion (Figure 2). ALK driver point mutations were identified in 3 patients (2%), and BRAF mutations in 10 (6.6%), where in 2 (1.3%) of them BRAF Class I mutations were identified. No driver alterations in the ROS‐1 gene were identified. Sites of metastatic lesions for the patients with alterations identified in EGFR, ALK, and BRAF are shown in Table S4 in the electronic Supporting Information.

Of the 79 patients (52.3%) who exhibited a TMB of 0 or more mutations per megabase (MB), the average TMB was 12.1 mutations/MB. Among these patients, 17 (21.5%) had a TMB of 16 or more mutations/MB, 20 (25.3%) had a TMB ranging from 10 to 15 mutations/MB, and 42 (53.2%) had fewer than 10 mutations/MB (Figure 2).

In samples from 82 (54.3%) patients, MSI was identified, corresponding to microsatellite stable (MSS) in 79 (96.3%) patients and to microsatellite instability‐high (MSI‐H) in 3 (3.7%) patients (Figure 2).

### 3.5 NSCLC Second Line Treatments as Per Standard of Care Versus FMI Comprehensive Genomic Profiling

**FIGURE 3:**
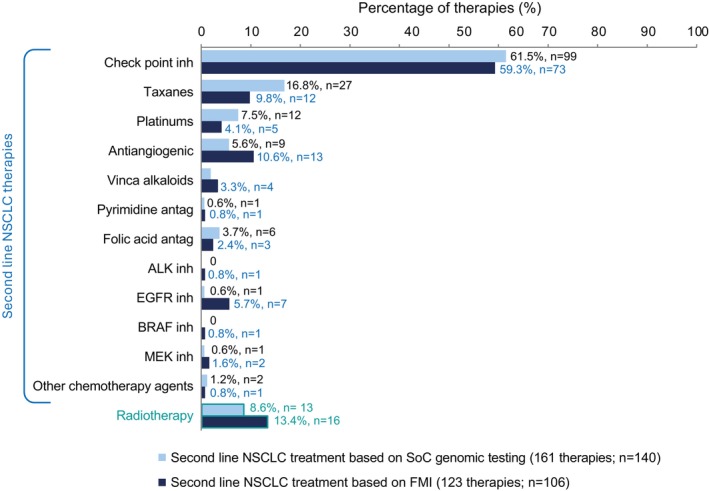
Comparison of second line treatment decisions as per SOC versus FMI report. CGP, comprehensive genomic profiling; FMI, foundation medicine information genetic CGP; inh, inhibitors; NSCLC, non‐small cell lung cancer.

Based on the SOC genetic profiling, a total of 161 s line therapies were to be administered in 140 patients, with a higher percentage of patients receiving checkpoint inhibitors (99 times, 61.5%), taxanes (27 times, 16.8%), and platinums (12 times, 7.5%) (Figure 3). Physicians decided that palliative radiotherapy would be administered to 13 (8.6%) patients.

Of the 106 (70.2%) patients that reported receiving second line treatment based on the FMI report, 94 (91.3%) were due to disease progression and 9 (8.7%) patients following the decision of the investigator. A total of 123 therapies other than radiotherapy were to be administered in 106 patients, with the most employed being checkpoint inhibitors (73 times, 59.3%), antiangiogenic (13 times, 10.6%), and taxanes (12 times, 9.8%) (Figure 3). After receiving the comprehensive genomic profiling provided by the FMI report, clinicians still administered radiotherapy to 16 (13.4%) patients (Figure 3).

Comparison between the choice of second line treatment using SOC genetic testing versus FMI showed that after FMI assessment, clinicians increased the use of radiotherapy, antiangiogenics, ALK, EGFR, MEK, and BRAF inhibitors and reduced platinums, taxanes, and folic acid antagonists.

### 3.6 Changes in the Decision of NSCLC Second Line Treatments After Receiving the FMI Report

For 14 (9.3%) patients, the second line treatment decision was changed after evaluating the FMI report (Table 3). None of these patients received radiotherapy, while checkpoint and EGFR inhibitors were administered in 5 (35.7%) patients each, change of previous chemotherapy regimen occurred for 2 (14.2%) patients, and ALK and BRAF inhibitors were used in 1 (7.1%) patient each. Table 4 shows the original therapy regimen for these patients. A total of 14.3% underwent resection surgery and 21.4% received radiotherapy. Regarding systemic therapies, most patients received combination regimens, predominantly 3 lines (57.1%), with the majority of therapies delivered as induction (67.6%).

**TABLE 3:** Changes in disease management induced by the analysis of the FMI report.

Second line NSCLC treatment decision after FMI report (*N* = 151)			
Maintained (*n* = 137)		Changed (*n* = 14)	
Reasons	*n* (%)^a^	Chosen treatments	*n* (%)^b^
FMI report did not provide any useful information	115 (76.2)	Check point inhibitors	5 (35.7)
The considered clinical trial is not available	11 (7.3)	EGFR inhibitors	5 (35.7)
		Change of chemotherapy	2 (14.2)
The considered treatment is not available	11 (7.3)	BRAF inhibitors	1 (7.1)
		ALK inhibitor	1 (7.1)

Abbreviations: FMI, foundation medicine Inc; NSCLC, non‐small cell lung cancer.

^a^ Percentages calculated over the total population (*n* = 151).

^b^ Percentages calculated over the total number of registered therapies (*n* = 14).

**TABLE 4:** Original regimen and FMI results for those patients with second‐line regimens changed after FMI (*n* = 14).

Parameter	*n* (Total *N* = 14)
*Original regimen*	
Resection surgery	2 (14.3)
Radiotherapy	3 (21.4)
*N*° of chemotherapy and other therapies^a^	
1	2 (14.3)
2	4 (28.6)
3	8 (57.1)
Therapy^b^	34 (100.0)
Induction	23 (67.6)
Maintenance	11 (32.4)
Type	
Folic acid antagonists	19 (55.9)
Platiniums	12 (35.3)
Vinca alkaloids	1 (2.9)
Antiangiogenic	1 (2.9)
Pyrimidine antagonists	1 (2.9)
*N*° of cycles, mean, SD	
Folic acid antagonists	7.3 (8.3)
Antiangiogenic	7.0.(−)
Vinca alkaloids	4.0 (−)
Pyrimidine antagonists	4.0 (−)
Platiniums	3.7 (1.2)
*FMI results*	
Therapeutical orientations = Yes^c^	13 (92.9)
Authorized therapy for NSCLC	13 (92.9)
Authorized therapy for another cancer different than NSCLC	13 (92.9)
Therapy in Clinical Trial	12 (85.7)
Genomic alterations identified	169 (100.0)
VUS	113 (66.9)
Driver	56 (33.1)
Actionable targets^a^	
EGFR	3 (21.4)
ALK	0 (0.0)
BRAF	2 (14.3)

*Note:* Data are shown *n* (%) unless otherwise indicated.

Abbreviations: CGPm, comprehensive genomic profiling; FMI, foundation medicine information genetic CGP; NSCLC, non‐small cell lung cancer; VUS, variants of unknown significance.

^a^ Analysis per number of patients.

^b^ Multiple response. Analysis of chemotherapy and other therapies is performed over a total of registers included (*n* = 34).

^c^ Multiple response.

Clinicians did not change their previous decision about the choice of second line treatment in 137 patients (90.7%), from which in 115 (76.2%) patients FMI did not provide any useful information, and in 22 (14.6%) patients the considered clinical trial or treatment was not available.

FMI report helped clinicians to choose the second line treatment of 41 (27.2%) patients (Table 5). Clinicians found gene alterations for which there was an approved drug in 39 (25.8%) patients. In 34 (87.2%) of these patients, somatic genetic alterations were not previously detected using the SOC procedures. Moreover, clinicians found that 44 (29.1%) patients carried somatic genetic alterations for which an off‐label drug existed, not previously detected by SOC in 43 (97.7%) of these patients. In addition, 13 (8.6%) patients were directed toward clinical trials based on the information provided by the FMI report, although only clinical trials for 2 of these patients were available (for patients with KRAS mutation in solid tumors and for patients with MET amplification). Overall, the disease prognosis of 34 (22.5%) patients was better defined when using FMI report information (Table 5).

**TABLE 5:** Disease management based on the FMI report analysis from the clinician perspective.

Clinician decision	
FMI report helped to choose 2nd line treatment, *n* (%)^a^	41 (27.2)
FMI report identified gene alterations with approved drugs, *n* (%)^a^	39 (25.8)
Not previously identified by SOC, *n* (%)^b^	34 (87.2)
FMI report identified gene alterations with off‐label drugs, *n* (%)^a^	44 (29.1)
Not previously identified by SOC, *n* (%)^c^	43 (97.7)
FMI report directed patient toward clinical trials, *n* (%)^a^	13 (8.6)
Available clinical trial^d^	2 (15.4)
FMI report helped to tune up disease prognosis, *n* (%)^a^	34 (22.5)

^a^ Percentages calculated over the total population (*n* = 151).

^b^ Percentages calculated over the total number of patients with identified gene alterations with approved drugs (*n* = 39).

^c^ Percentages calculated over the total number of patients with identified gene alterations with off‐label drugs (*n* = 44).

^d^ Percentages calculated over the total number of patients directed toward clinical trials (*n* = 13).

## 4 Discussion

The present study showed that FMI technology can identify actionable driver mutations otherwise missed by SOC methodology. Our findings also highlight the value of FMI as a diagnostic tool to identify matched targeted therapies, including approved and off‐label therapies and therapies in clinical trials, and its potential to change SOC clinical management of NSCLC patients.

The diagnosis of NSCLC and consequent treatment decisions [14] starts with pathological diagnosis that should be made according to the World Health Organization (WHO) and ESCAT classifications [12, 13, 32, 33], followed by therapy‐predictive biomarker testing. Current guidelines advise two non‐exclusive but complementary testing approaches, one for immuno‐oncology therapy biomarker testing (PD‐1/PDL‐1 expression) and the other for the detection of actionable oncogenic alterations (EGFR, KRAS, BRAF, ERBB2, MET, ALK, ROS‐1, RET, NTRK1/2/3 fusions and PDL‐1 expression) [7, 8, 9, 10, 11, 34]. Current SOC genomic testing uses a combination of individual standalone testing methods including PCR‐based assays, Sanger sequencing, FISH, IHC, and multi‐gene testing, including NGS of various sorts [14, 35, 36]. This practice will be driven by the availability of treatments and will vary widely between different health systems, region within in the same country, and from facility to facility [14, 36, 37, 38, 39]. Those differences can lead to discrepancies in testing accessibility and subsequent treatment options for patients [36]. This heterogeneity of methodologies is confirmed in the current study, where the chosen technique when testing for EGFR, ALK, BRAF and ROS1 genetic alterations differed depending on the target gene. The use of large comprehensive NGS was still limited in Spain at the time the study was carried out. Expert opinion suggests that predictive biomarker testing should shift toward large‐panel formats [6]. This trend is evident in current practice, where NGS for advanced and/or metastatic NSCLC patients has a funded indication through the public health system. Large‐panel testing, such as FMI, allows for the detection of all currently targetable molecular aberrations. It also supports molecular testing for emerging predictive and prognostic biomarkers for both EMA‐approved and experimental therapies, a comprehensive approach which will ensure equal access to available systemic treatments for all patients [6]. Indeed, our results demonstrate that the FMI platform detected more genomic alterations in actionable genes with ESCAT level I, and in a higher number of patients, compared to genetic tests performed according to SOC.

Prevalence of activating alterations in the EGFR gene is among 10% to 35% of patients with NSCLC [14, 40], fusion genes involving ALK account for about 2% to 10%, ROS1 fusion genes occurs in around 1% to 4% of the same testing population, and V600 BRAF family mutations are found in up to 2% of cases [14, 25, 40, 41, 42]. SOC genetic tests identified in the current study two patients with BRAF V600E mutations, and 8 patients with mutations in BRAF, KRAS, STKK11, and TP53, whereas EGFR‐activating alterations, ALK rearrangements, and ROS‐1 fusion were not found. On the other hand, FMI identified a total of 1582 genomic alterations, of which 542 (34.3%) were driver mutations; 9 (6.8%) patients with alterations in the EGFR gene, including exon 19 deletions, 3 patients (2.3%) with ALK mutations, and 10 (7.6%) patients with mutations in the BRAF genes, including class I mutations. Intriguingly, none of these patients previously tested using SOC methods showed any of these alterations. In addition, driver alterations in ERBB2, MET, and RET, but not HER2, were identified in patients using FMI. When comparing biopsy types, our results suggest that tissue‐based genomic profiling identified therapeutic opportunities and more alterations than liquid biopsy, with significant differences in the likelihood of identifying actionable genomic findings and receiving therapeutic orientations.

Alterations of key genes were therefore underrepresented when SOC methods were used compared to FMI. This is in line with several previous reports [19, 29, 36, 43]. Schrock et al. were able to identify alterations in the EGFR gene of NSCLC patients using CGP which were previously missed according to the SOC genetic panels [40]. Ali et al. showed that 65% of patients with actionable ALK rearrangements identified by CGP were missed by the single‐gene testing method FISH [44]. In another study, 65% of NSCLC tumors that seemed to be negative by non‐NGS testing were found to have actionable genomic alterations [45]. In the same line, non‐inferiority of FMI compared to FDA‐approved companion diagnostic devices has been shown in seven clinical concordance studies [27, 41].

The patients from this study were not treated with targeted therapies as first line treatment according to the selection criteria. Prevailing clinical practice for patients with locally advanced (stage III) NSCLC is to check its suitability for resection surgery, and 40 (26.5%) patients underwent surgery as first line therapy. In line with the recommendation of chemoradiotherapy for unresectable stage III NSCLC, 35 (23.2%) patients received radiotherapy and a total of 167 (43.5%) chemotherapies, including platinums, taxanes, vinca alkaloids, and pyrimidine antagonists. For those patients without driver mutations identified by SOC who have not received prior immunotherapy, anti PD‐1/PD‐L1 immune therapy is recommended irrespective of PD‐L1 expression [5, 14]. In the current study and following ESMO and ASCO recommendations, identification of patients with EGFR, ALK, and BRAF targetable driver alterations by FMI matched with the administration of targeted therapies: checkpoint and EGFR inhibitors were administered in 5 (35.7%) patients each, and ALK and BRAF inhibitors were used in 1 (7.1%) patient each. Still, most of the patients did not have any of the recommended targetable driver mutations, and, given that these patients were not previously treated with immune therapies, immune checkpoint inhibitors were the most used second line therapies (59.3%).

High TMB is being validated with promising results as a biomarker for response to immunotherapy and, in fact, the FDA has approved the PD‐1 inhibitor, pembrolizumab, as a therapy for all solid tumors with TMB equal to or greater than 10 mutations/MB as measured by the FoundationOne CDx assay [30, 31]. In the current study, 17 (21.5%) patients had ≥ 16 mutations/MB and 20 (25.3%) patients had between 10 and 15 mutations/MB. These patients could have been candidates for immunotherapy according to their high TMB [46, 47]. Concerning microsatellite instability, this study identified only 3 patients with MSI‐H by FMI, in agreement with a previous report that the incidence of MSI‐H has been reported as low in lung cancer [48].

FMI identified mutations with designated therapies in 116 patients. Using this data, clinicians found 44 patients (29.1%) with gene alterations associated with off‐label drugs, and 13 (8.6%) patients were directed toward clinical trials. FMI has the potential to improve the probability of success of clinical trials by selecting adequate patients by biomarkers and changing SOC clinical management of NSCLC patients. After consideration of the FMI reports by the clinicians, the second line treatment decision based on SOC testing was changed in 14 (9.3%) patients. We can speculate that these patients redirected to targeted therapies will have had better clinical outcomes based on the known improved clinical outcomes of patients treated with matched therapies [41, 42, 44, 45, 49, 50].

Some limitations should be considered in the interpretation of the study data, mainly arising from its observational nature. This study was designed to collect information available in routine clinical practice, and thereby, the validity of study results was limited by the information available and the presence of missing data. In addition, patients were included between October 2018 and July 2019, with a maximum follow‐up period of 24 months, providing a snapshot of the clinical setting in Spain approximately 3 years ago. It showed the benefit of implementing tools such as FMI genomic profiling, and fortunately, the use of such methodology in routine clinical practice in Spain has increased significantly in recent years. To the best of our knowledge, our series of 180 patients with advanced NSCLC comprised the first study focused on the Spanish population directly assessing the impact of FMI analysis on SOC planned second line therapy in advanced NSCLC. Since the study included a total of 12 centers located in different regions throughout Spain, our results could be generalizable to the Spanish population with advanced NSCLC. Nevertheless, generalizability may be limited in settings with different access to CGP, distinct reimbursement policies, or variable diagnostic infrastructure. It should also be noted that the results primarily apply to patients with advanced NSCLC that have not been treated with targeted therapies as first line treatment. Moreover, we acknowledge that the study was designed to evaluate the impact of CGP on second‐line treatment decision‐making, and patient survival and response outcomes were not evaluated.

Another potential limitation of incorporating CGP such as Foundation Medicine assays in clinical practice is the turnaround time (TAT) required to obtain results, which can range from 7 to 21 days depending on the platform and sample type [51]. Prolonged TAT may limit timely access to targeted therapies or clinical trials, particularly in aggressive or rapidly progressing tumors, emphasizing the need for optimized workflows and rapid sequencing methods in precision oncology. Specifically, for the LungONE study, TAT did not have a direct clinical impact since patients were already receiving active first‐line treatment without clinical progression at the time of analysis, and CGP results were intended to guide second‐line therapy decisions. Externalized CGP approaches may also encounter other challenges, such as sample shipping, pre‐analytical conditions of tissue, DNA quality and yield, and tumor content limits required for this FMI assay, that contribute to failure rates in the process. Even with low analytical failure rates and high reproducibility in FMI, cases may be rejected by the laboratory due to insufficient tumor content, DNA preservation, or low tumor fraction. On the other hand, the cost of the test must be taken into account as CGP is more commonly used in academic settings with the goal of enrolling patients in clinical trials.

## 5 Conclusions

In conclusion, this analysis demonstrates the benefit of FMI genomic profiling identifying actionable driver mutations otherwise missed by SOC methodology. Our findings also highlight the value of FMI as a diagnostic tool to identify matched targeted therapies, including off‐label therapies and therapies in clinical trials. Using FMI data, clinicians directed patients toward clinical trials and changed SOC clinical management of several NSCLC patients. This study shows that FMI can contribute to the improvement of personalized medicine.

## Author Contributions

**F. Rojo:** conceptualization (lead); investigation (lead); methodology (lead); writing – original draft preparation (lead); writing – review and editing (equal). **E. Felip:** conceptualization (equal); investigation (equal); methodology (equal); writing – original draft preparation (supporting); writing – review and editing (equal). **O. Juan‐Vidal:** conceptualization (equal); investigation (equal); methodology (equal); writing – original draft preparation (supporting); writing – review and editing (equal). **R. García‐Campelo:** conceptualization (equal); investigation (equal); methodology (equal); writing – original draft preparation (supporting); writing – review and editing (equal). **D. Aguiar‐Bujanda:** conceptualization (equal); investigation (equal); methodology (equal); writing – original draft preparation (supporting); writing – review and editing (equal). **J. Terrasa:** conceptualization (equal); investigation (equal); methodology (equal); writing – original draft preparation (supporting); writing – review and editing (equal). **R. López Castro:** conceptualization (equal); investigation (equal); methodology (equal); writing – original draft preparation (supporting); writing – review and editing (equal). **A. Calles Blanco:** conceptualization (equal); investigation (equal); methodology (equal); writing – original draft preparation (supporting); writing – review and editing (equal). **A. Paredes:** conceptualization (equal); investigation (equal); methodology (equal); writing – original draft preparation (supporting); writing – review and editing (equal). **R. Bernabé:** conceptualization (equal); investigation (equal); methodology (equal); writing – original draft preparation (supporting); writing – review and editing (equal). **I. Barneto:** conceptualization (equal); investigation (equal); methodology (equal); writing – original draft preparation (supporting); writing – review and editing (equal). **Luis García‐Palacios:** conceptualization (lead); funding acquisition (lead); investigation (lead); methodology (lead); writing – original draft preparation (lead); writing – review and editing (equal). **J. De Castro Carpeño:** conceptualization (lead); investigation (lead); methodology (lead); writing – original draft preparation (lead); writing – review and editing (equal).

## Funding

This study was sponsored by Roche Farma, Spain.

## Ethics Statement

The study is in accordance with the Declaration of Helsinki and Good Clinical Practice Guidelines and applicable regulatory requirements. The study protocol was approved by the Independent Ethics Committee (IEC) of Hospital Universitario de Gran Canaria Dr. Negrín (CEIm code 2018‐185‐1).

## Consent

Written informed consent was obtained from all patients to participate in the study before their enrollment.

## Conflicts of Interest

F.R.: Advisory board: Roche, Astra Zeneca, Pfizer, Menarini‐Stemline, Lilly, Daiichi‐Sankyo, Abbvie, BMS, Merck Sharp & Dohme, Merck, GSK, Novartis, Sophia Genetics, Janssen, Agilent, Astellas; Speaker honoraria: Roche, Astra Zeneca, Pfizer, Menarini‐Stemline, Lilly, Daiichi‐Sankyo, Abbvie, BMS, Merck Sharp & Dohme, Merck, GSK, Novartis, Sophia Genetics, Janssen, Agilent, Astellas; Research funding: Roche, Pfizer, Astra Zeneca, Menarini‐Stemline. E.F.: Advisory board: Abbvie, Amgen, AstraZeneca, Bayer, Boehringer Ingelheim, BMS, Daiichi Sankyo, F. Hoffmann‐La Roche, Genmab, Gilead, GSK, ITeos Theraputics, Janssen, Johnson & Johnson, Merck Sharp & Dohme, Novartis, Pierre Fabre, Pfizer, Regeneron, Turning Point; Speaker honoraria: Amgen, AstraZeneca, BMS, Daiichi Sankyo, Eli Lilly, F. Hoffmann‐La Roche, Johnson & Johnson, Genentech, Gilead, Janssen, Medical Trends, Medscape, Merck Serono, Merck Sharp & Dohme, Novartis, Peervoice, Pierre Fabre, Pfizer, Regeneron, Touch Oncology; Board of Director role: Grifols; Financial support for meeting attendance and/or travel: AstraZeneca, Janssen, Roche. O.J.‐V.: Honoraria or Advisory role: Bristol‐Myers Squibb, Roche/Genentech, AstraZeneca, Pfizer, Eli Lilly, Takeda, Janssen, Novartis; Travel, accommodations, expenses: Takeda, AstraZeneca, Roche/Genentech, Pfizer, Merck Sharp & Dohme, Roche/Genentech. R.G.‐C.: Advisory board: Amgen, AstraZeneca, Bayer, BMS, Boehringer Ingelheim, F. Hoffmann‐La Roche, Janssen, Lilly, Merck Sharp & Dohme, Pfizer, Sanofi, Takeda, Pfizer; Research funding: Amgen, AstraZeneca, Bayer, BMS, Boehringer Ingelheim, F. Hoffmann‐La Roche, Janssen, Lilly, MSD, Pfizer, Sanofi, Takeda, Pfizer; Personal: Amgen, AstraZeneca, Bayer, BMS, Boehringer Ingelheim, F. Hoffmann‐La Roche, Janssen, Lilly, Merck Sharp & Dohme, Pfizer, Sanofi, Takeda, Pfizer. R.L.C.: Speaker honoraria: Roche, Aristo, Pierre Fabre, Pfizer, AstraZeneca, Novartis; Advisory Board: Roche, Merck Sharp & Dohme, BMS, Pierre Fabre, AstraZeneca, Amgen, Novartis; Travel and inscription to medical meetings: MSD, Roche, Janssen. A.C.B.: Advisory Board: AstraZeneca, Boehringer Ingelheim, Bristol‐Myers Squibb, Janssen, Lilly, Merck Sharp & Dohme, Novartis, Pfizer, Regeneron, Roche, Sanofi, Takeda; Speaker honoraria: Boehringer Ingelheim; Invited Speaker: PharmaMar; Research Grant, Institutional, Financial interest, Drug‐only for Investigator‐initiated trial: Merck Sharp & Dohme; Personal: AstraZeneca, Bayer, Bristol‐Myers Squibb, Janssen, Lilly, Sharp & Dohme, Novartis, Pfizer, PharmaMar, Regeneron, Roche, Sanofi, Takeda. A.P.: Advisory Board: Roche, AstraZeneca, Pfizer, Eli Lilly; Travel, accommodations, expenses: Pfizer; Personal: Roche, AstraZeneca, Pfizer, Eli Lilly. R.B.: Advisory board: Takeda, Roche, BMS, AstraZeneca; Speaker honoraria: Roche, BMS, Pfizer, Merck Sharp & Dohme, Amgen, Takeda, AstraZeneca. L.G.‐P.: is an employee of Roche Farma S.A. J.D.C.C.: Advisory Board: AstraZeneca, Bristol‐Myers Squibb, Merck Sharp & Dohme, Pfizer, Roche, Takeda, GlaxoSmithKline, Janssen Oncology, Lilly, Amgen, Beigene, Daiichi Sankyo/Astra Zeneca, Regeneron, Pierre Fabre, Gilead Sciences; Travel, accommodations, expenses: AstraZeneca, Merck Sharp & Dohme, Pfizer, Roche, Daiichi Sankyo/Astra Zeneca. The other authors declare no conflicts of interest.

## Supporting information


**Appendix S1:** cam471790‐sup‐0001‐supinfo1.docx.
**Table S1:** NSCLC first line treatments.
**Table S2:** Additional genetic alterations (in genes distinct from ALK, EGFR, ROS‐1, BRAF and PD‐L1).
**Table S3:** Comparison between FMI testing of tissue versus liquid biopsy.
**Table S4:** Sites of metastatic lesions in patients with driver mutations.
**Figure S1:** Patients best response* after first line treatment.

## Data Availability

The data that support the findings of this study are available on request from the corresponding author. The data are not publicly available due to privacy or ethical restrictions.
