# Supplementary material for: Second Line Treatment Decision as Per Standard of Care or Foundation Medicine in Patients With Locally Advanced or Metastatic Non‐Small Cell Lung Cancer
Source: Cancer Med. 2026 Apr 12;15(4):e71790. doi: 10.1002/cam4.71790 (PMC13071178; doi:10.1002/cam4.71790)
Supplement: Supplementary file 1 — Appendix S1: cam471790‐sup‐0001‐supinfo1.docx. Table S1: NSCLC first line treatments. Table S2: Additional genetic alterations (in genes distinct from ALK, EGFR, ROS‐1, BRAF and PD‐L1). Table S3: Comparison between FMI testing of tissue versus liquid biopsy. Table S4: Sites of metastatic lesions in patients with driver mutations. Figure S1: Patients best response* after first line treatment. [file CAM4-15-e71790-s001.docx]

**Supplementary material**

**Supplementary - Table S1: NSCLC first line treatments**

| **Treatment regimen** |  |  |
| --- | --- | --- |
| **NSCLC first line therapies^1^** |  |  |
| Folic acid antagonists, n (%) | | 172 (44.8) |
| Cycles, mean (S.D.) | | 8.8 (9.9) |
| Induction (n)/Maintenance (n) | | 92/80 |
| Platinums, n (%) | | 138 (35.9) |
| Cycles, mean (S.D.) | | 4.1 (1.2) |
| Induction (n)/Maintenance (n) | | 138/0 |
| Antiangiogenic, n (%) | | 41 (10.7) |
| Cycles, mean (S.D.) | | 13.2 (17.1) |
| Induction (n)/Maintenance (n) | | 17/24 |
| Taxanes, n (%) | 18 (4.7) |  |
| Cycles, mean (S.D.) | 4.7 (1.1) |  |
| Induction (n)/Maintenance (n) | 18/0 |  |
| Vinca alkaloids, n (%) | 9 (2.3) |  |
| Cycles, mean (S.D.) | 12.9 (17.9) |  |
| Induction (n)/Maintenance (n) | 9/0 |  |
| Pyrimidine antagonists, n (%) | 2 (0.5) |  |
| Cycles, mean (S.D.) | 4.0 (0.0) |  |
| Induction (n)/Maintenance (n) | 2/0 |  |
| Check point inhibitors, n (%) | 1 (0.3) |  |
| Cycles, mean (S.D.) | 3.0 (-) |  |
| Induction (n)/Maintenance (n) | 1/0 |  |
| Other chemotherapy agents not aforementioned, n (%) | 3 (0.8) |  |
| Cycles, mean (S.D.) | 8.0 (8.7) |  |
| Induction (n)/Maintenance (n) | 2/1 |  |
| **Surgery**, n (%)^2^ | | 40 (26.5) |
| **Radiotherapy**, n (%)^2^ | | 35 (23.2) |
| Cycles, mean (S.D.) | | 9.1 (8.3) |

(1) Percentages calculated over the total number of registered therapies other than radiotherapy (n=384); (2) Percentages calculated over the total population (n=151).

**Supplementary - Table S2: Additional genetic alterations (in genes distinct from ALK, EGFR, ROS-1, BRAF and PD-L1)**

| **Gene** | **n (%)*** |
| --- | --- |
| BRAF | 1 (9.1) |
| KRAS | 8 (72.7) |
| STK11 | 1 (9.1) |
| TP53 | 1 (9.1) |

* Percentage calculated for n=11 genetic alterations in 8 patients.

**Supplementary – Table S3: Comparison between FMI testing of tissue *versus* liquid biopsy.**

| Parameters | **Sample type (Total N=151)** | | ***p* value** |
| --- | --- | --- | --- |
|  | Tissue  (Foundation ONE) | Blood  (Foundation ONE Liquid) |  |
| **Proportion of the study population** | 102 (67.5) | 49 (32.5) |  |
| **Therapeutical orientations = Yes*** | 90 (88.2) | 26 (53.1) | *p*<0.001^1^ |
| Therapy in Clinical Trial | 88 (97.8) | 24 (92.3) |  |
| Authorised therapy for another cancer different than NSCLC | 75 (83.3) | 24 (92.3) |  |
| Authorised therapy for NSCLC | 69 (76.7) | 13 (50.0) |  |
| **Genomic alterations identified**  *Total = 1582* | 1390 | 192 | *p*<0.001^1^ |
| Driver | 455 (32.7) | 87 (45.3) |  |
| VUS | 925 (67.3) | 105 (54.7) |  |
| **Actionable targets**** |  |  |  |
| EGFR | 6 (6.3) | 3 (8.1) | *p*=0.710^2^ |
| ALK | 3 (3.2) | 0 (0.0) | *p*=0.559^2^ |
| BRAF | 10 (10.5) | 0 (0.0) | *p*=0.061^2^ |

Data are shown n (%).

^1^Chi squared; ^2^Fisher Exact test.

*Multiple response; percentage calculated over all patients with therapeutical orientations (n=90 for tissue and n=26 for blood sample type).

** n=132 (n=95 for tissue and n=37 for blood sample type)

*NSCLC= Non-small Cell Lung Cancer; VUS= Variants of Unknown Significance.*

**Supplementary - Table S4: Sites of metastatic lesions in patients with driver mutations.**

| **Patients with altered gene=** | **Metastatic lesions,** *n (%)* | | | |
| --- | --- | --- | --- | --- |
|  | **Lymph nodes** | **Lung/Pleura** | **Bone** | **Brain** |
| EGFR (n=9) | 2 | 2 | 2 | 1 |
| ALK (n=3) | - | 1 | - | 2 |
| BRAF (n=10)* | 1 | 3 | 4 | 1 |

* One patient registers metastatic lesion in “lymph nodes, bone” in the eCRF.

**Supplementary – Figure S1: Patients best response* after first line treatment.**


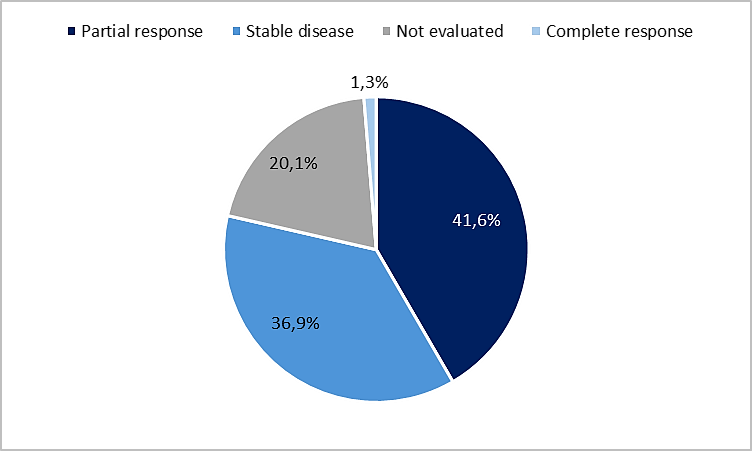


**Missing data: n=2, percentages*
